# Perception and knowledge of learners about the use of 3D technologies in manual therapy education – a qualitative study

**DOI:** 10.1186/s12909-023-04497-6

**Published:** 2023-07-17

**Authors:** Kesava Kovanur Sampath, Ashokan Arumugam, Esra Yaghi, Emma Fairs, Patrea Andersen

**Affiliations:** 1 Centre for Health and Social Practice, Waikato Institute of Technology, 51, Akoranga Road, Hamilton, New Zealand; 2 Department of Physiotherapy, College of Health Sciences, University of Sharjah, P.O.Box 27272, Sharjah, United Arab Emirates; 3 Neuromusculoskeletal Rehabilitation Research Group, RIMHS–Research Institute of Medical and Health Sciences, University of Sharjah, P.O.Box: 27272, Sharjah, United Arab Emirates; 4 Sustainable Engineering Asset Management Research Group, RISE-Research Institute of Sciences and Engineering, University of Sharjah, P.O.Box: 27272, Sharjah, United Arab Emirates; 5 Department of Physiotherapy, Manipal College of Health Professions, Manipal Academy of Higher Education, Manipal, Karnataka India; 6 Ara Institute of Canterbury, Christchurch, New Zealand; 7 School of Nursing, Midwifery and Paramedicine, University of the Sunshine Coast, Sippy Dow, QLD 4556 Australia; 8 School of Nursing, Midwifery and Social Science, CQ University, Queensland, 4702 Australia

**Keywords:** Manual therapy, Education, Physiotherapy, Osteopathy, 3D technology, Virtual reality

## Abstract

**Objectives:**

Manual therapy is a specific hands-on approach used and taught by various professions such as physiotherapy and osteopathy. The current paradigm of teaching manual therapy incorporates the traditional ‘See one, do one, teach one’ approach. However, this ‘teacher centred’ approach may not enable learners to develop the complex clinical skills of manual therapy. In this context, 3D technologies such as virtual reality may facilitate the teaching and learning of manual therapy. Hence the aim of the current study was to investigate the perception, knowledge and attitude of manual therapy learners about the use of 3D technologies in manual therapy education.

**Methods:**

An exploratory qualitative research design using semi-structured interviews was used in this study. A total of ten manual therapy (5 physiotherapy and 5 osteopathic) students (mean age = 32; 80% female) enrolled in an appropriate physiotherapy or osteopathic degree provided by a New Zealand recognized institution (e.g., university or polytechnic) participated in this study. Data saturation was achieved after 10 interviews (average duration: 35 min) that provided thick data. A thematic analysis was used for data analysis.

**Results:**

Six factors were identified which appeared to influence participants’ perception of role of technology in manual therapy education. These were (1) the sufficiency of current teaching method; (2) evolution as a learner (a novice to an expert); (3) need for objectivity; (4) tutor feedback; (5) knowledge and (6) barriers and enablers. These six factors influenced the participants’ perception about the role of 3D technologies in manual therapy education with participants evidently taking two distinct/polarized positions (‘no role’ (techstatic) versus a ‘complete role’ (techsavvy)).

**Conclusion:**

Although 3D technology may not replace face-to-face teaching, it may be used to complement the traditional approach of learning/teaching to facilitate the learning of complex skills according to the perceptions of manual therapy learners in our study. The advantage of such an approach is an area of future research.

**Supplementary Information:**

The online version contains supplementary material available at 10.1186/s12909-023-04497-6.

## Background

Manual therapy (MT) is a specific hands-on approach used by various professions such as physiotherapy and osteopathy. Learners of MT are required to develop complex clinical skills such as clinical reasoning, manual/physical assessments, palpation and patient management which include skilled hands-on treatment [1]. The process of learning clinical MT skills usually incorporates the traditional ‘See one, do one, teach one’ approach. This ‘teacher centred’ approach consists of students observing an expert clinician/tutor performing the techniques on a student, a plastic anatomical model, or a patient [2, 3]. The underlying assumption is that learners become increasingly independent after observing an expert clinician or teacher [4]. The teacher/expert then proceeds to check the learner’s technique and provide feedback. By providing feedback and guidance, it is believed that the ‘see one, do one’ approach may enable the learners to grasp the varied physical examination, palpation and treatment skills [5].

Nevertheless, this approach has been criticized as an inadequate method in maintaining required patient safety standards [4, 6]. This is due to lack of supervision, reflection on action, performance evaluation and structured feedback [7]. Studies show that between 28 and 42% of medical residents felt inadequately trained to safely perform a medical procedure alone for the first time [8, 9]. These could be attributed to the traditional (see one do one) teaching methods [9]. A meta-analysis [10] compared the effectiveness of traditional (see one do one) methods of clinical medical education versus simulation based medical education with deliberate practice. The findings from the study clearly demonstrated that not only was student performance better in the simulated group, benefits were observed in long-term retention of skills [10].

Three dimensional (3D) digital technologies such as virtual reality (VR), augmented reality (AR) and mixed reality technologies have been used in several applied fields including teaching and learning [11]. While in a VR environment, the user is completely immersed [11], an AR system combines or “supplements” real world objects with virtual objects or superimposed information [12]. Further, some VR systems have in-built haptic (sense of touch) devices (e.g., the Geomagic, the Phantom Omni). The addition of haptic feedback in VR environments creates more realistic scenarios, while providing trainees with a safe environment in which they can develop their skill [13]. Few haptic devices are specifically designed to enable the user to grasp virtual objects and provide varying degrees of freedom [14, 15] that could be adapted for MT education.

MT students are taught to do manual examination with their hands which includes soft tissue palpation (abnormal tissue resistance), joint range of motion and pain provocation in many musculoskeletal disorders (e.g., spinal segmental dysfunction) [16–18]. Although, the criterion validity of this type of manual examination has been proven [17–20]; the inter-rater reliability and validity of many MT techniques need further substantiation. Moreover, the ongoing COVID 19 pandemic and associated lockdown measures necessitated the use of distance/remote/online/virtual or hybrid learning by most educational institutions [21]. For MT education, this has resulted in an unprecedented challenge of teaching an essentially “hands-on” content through an “online” method. Even so, learning the complex MT skills online could be challenging for MT learners and such methods may undermine the need of patient safety skills.

Evidence indicates that 3D technologies in educational settings can improve task completion times, increase engagement, lead to fewer errors, and improve student’s motivation to learn [22–24]. The immersive experience of VR has been shown to be effective in understanding age-related health problems and increasing empathy of medical and other healthcare students for older adults [25]. Furthermore, learning style theories suggest that there are three main ways to learn: auditory, visual and kinaesthetic learning styles [24]. In this context, 3D technologies such as VR may facilitate all three of these learning styles in one application [24, 26, 27]. Hence, such technologies may help MT learners to grasp the complex and intricate skills of MT. A recent scoping review [28] pointed out that although there are no 3D technology applications that specifically serve the needs of MT education, but applications are available that can be readily used or potentially adapted to train MT skills. For example, Howell et al. developed a virtual haptic back (VHB) [29] emulating the contours and the tissue textures of the human back to osteopathic medical students. Their results showed that both accuracy and speed of palpation by those students improved from using VHB, thereby resulting in the mastery of palpatory process [29]. Creating 3D educational applications such as VR can be tedious and/or expensive for all stakeholders (the developers, the institution, and the learners). Hence, it is important to investigate whether these technologies are perceived to be useful or not by MT learners themselves. This exploratory qualitative research aims to address this gap in the literature.

### Research question

- What is the perception and knowledge of MT learners about the use of 3D technologies in MT education?

### Aim/objectives

The aims of the current qualitative study are to: 1) investigate the perceptions and knowledge of MT leaners about the use of 3D technologies in MT education and (2) explore the barriers and enablers for using 3D technologies as part of MT education.

## Methods

We followed the Consolidated Criteria for Reporting Qualitative Studies guidelines for reporting this qualitative research [30]. Ethical approval for the study was provided by Waikato Institute of Technology’s Research Ethics Committee (WTLR32200721; 19/08/21). All participants signed an informed consent sheet prior to participation.

### Study design

An exploratory qualitative research design using semi-structured interviews was used in this study. The exploratory approach was considered appropriate to enable an in-depth understanding of the use of 3D technology by MT learners [31].

### Participants

A total of 10 students enrolled in an appropriate MT course provided by a New Zealand recognized institution such as a polytechnic or a university participated in this study. The MT course was delivered under two different settings: (1) full-time on campus and (2) a ‘block and blended’ approach where students were on campus during block weeks and did remote learning at other times. An email was sent with study details to various membership bodies and institutions that teach MT and convenience/purposive sampling was used to recruit participants. The demographic information of participants is provided in Table 1.

**Table 1:** Participant demographics

Mean Age	32
Gender	2 Males 8 Females
Profession	5 Physiotherapy Students 5 Osteopathy Students
Year of Study	5 Year three 2 Year two 2 Year one 1 Year four
Learning Setting	5 Face to face 5 Blend and block

### Data collection

Data collection methods involved semi-structured interviews via Zoom between August 2021 to February 2022. These were mostly conducted by the primary investigator (PI). As some of the participants were known to the PI, a research assistant conducted interviews with these participants. An interview guide (Additional file 1) was developed and used based on professional experience and findings from the literature [11, 28, 32–34]. The interview guide was piloted before data collection to ensure clarity. The researcher was always available to answer any questions from participants and provide clarifications where required. The participants were encouraged to ask any questions/doubts at any stage during the interview to reduce ambiguity. The average duration of the interviews was 35 min. Field notes were taken to facilitate data analysis. All interviews were recorded and transcribed verbatim. A diversity of viewpoints was captured that enabled achieving data sufficiency/saturation after the tenth interview.

### Data analysis

A thematic analysis was used for data analysis [35]. Each interview set was initially analysed independently by two investigators (KSK and EY). Initial coding was undertaken and assisted by NVivo V.10 qualitative analysis software. Category and theme development from the initial codes was an iterative/analytical process [35]. This involved reading and re-reading the transcripts/memos/field notes looking for patterns in the data (such as differences and commonalities). Memos were written throughout the analytical process that facilitated reflexivity by making it explicit any a priori biases of the researchers, thereby contributing to the credibility of the research. The themes generated were assessed by a third investigator (member checking) for plausibility and explanatory values against the transcripts. The key themes, subthemes and supporting quotes that resulted in the five factors are presented in Table 2. Finally, categories (factors) along with the themes were developed from the student interviews.

**Table 2:** Themes, sub-themes and supporting quotes of participant’s perception and knowledge about 3D technologies in manual therapy education

**Category**	**Themes**	**Sub-Themes**	***Quotes***
**Sufficiency of Current Teaching Methods**	**Theme 1 Only way to teach MT**	**Learning with hands**	*“I feel like it is sufficient…for us to be a better therapist [and] to learn better with our hands” (P2)* *"Yeah I think like pretty much teaching this way is perhaps the best way to teach hands-on papers" (P8)*
		**No other way to do it**	*“I can’t think of another way of doing it than the way they do it. To me what they do is great but it’s maybe because I’ve never thought about [it]”. (P6)* *"I have done a health degree before. I'm not aware of any other teaching methods for a practical course like osteopathy" (P10)*
	**Theme 2** **Established routine**	**Having a Routine**	*“[The] actual practical part side of it I think is really good, like they have a routine. They’ll show us a technique or like a special test or whatever they do, and then we’ll go away in our little groups and practise that, and I think that’s really like effective. Then the teachers come round and like adjust us if needed and just like help us and give us like random tips and stuff” (P7)* *"They break it into categories. For example, the cervical we would learn about the anatomy and then would go into the objective testing which may involve massage techniques or range of motion and then maybe even manipulations. Then talking about red flags and whatnot, when is the most appropriate time to use it. That is how it is structure and that structure is the same for other regions of the body" (P5)*
		**Knowing what to expect**	*"The practical sessions are structured, so we kind of know what to expect" (P8)* *"There are two different tutors. If one isn’t working you can speak to the other one and they might have a different approach for you to try. This is the same during every session" (P10)*
	**Theme 3** **Techer centric**	**Unhelpful for learners**	*“I’d like to say yes but no, not really. I almost feel as if a lot of it is just left up to you to try and work out if you’re doing it right. The tutor can’t feel exactly what you’re doing. They can only look and think it does look you’re pressing to hard or light or whatever. It would be really good if there was some way that you could actually have some sort of measurement of exactly how you do it or whether you’re doing it right or what you’re feeling is the correct thing” (P9)* *"The teachers do a bit, I mean maybe like twenty percent of my learning, and I would go home and watch a lot of videos and read a lot text books to get that visual visualisation of what’s under my hands, really like well, you know, so I can actually see it" (P4)*
		**Content dumping**	*“The way it’s been working is we have these eight hour long days and we have to cram/study all this information in the morning, have lunch, and then come back and cram the rest of the knee and maybe even the whole lower limb for the rest of the day and we just go away at night just feeling so overwhelmed, and then write an essay on a completely different subject” (P1)* *It could be more efficient. Why I say that is because when we are learning manual therapy techniques in class we don’t have that much opportunity to actually go and liaise with our other classmates, out lecturers, because it’s only two week block courses and then we come back and do our own studies, assessments and whatnot" (P5)*
**Evolution as Learner**	**Theme 4** **Palpation requirements**	**Gross Motor skills**	*“At the moment because I’m a first year I’m getting comfortable with touching people and making sure that when you’re holding their leg they feel like she’s got me. That’s what we’re working on at the moment”. (P6)* *"Some techniques are easily understood. for example, we had a sessions on elbow joint assessment. The palpation requirements were not as complex as some other joints." (P4)*
		**Subtle Palpation**	*“I’m finding it really difficult because you don’t know what you’re trying to feel. Don’t know what you’re feeling for and trying to translate a description into trying to work out what I’m feeling. I’m actually struggling with that a little bit. Generally the more obvious techniques are great but when it comes to really subtle palpation it’s really difficult to try and understand what you’re meant to be feeling for” (P9)* *"At the moment we’re trying to feel the motility of the gall bladder and it’s hard to explain. It’s like this corkscrew movement thing. You’ve got to be very careful. You sometimes can’t actually feel it and not everyone can feel it. That’s me. It’s difficult because they try and describe it but you’re not quite sure what you’re actually meant to be feeling" (P10)*
		**Previous MT experience**	*“ I have worked in massage now for five years, so I’ve got a lot of palpatory hands on experience but it doesn’t make me like an osteopath or anything, but for some of the school leavers, they come straight out of school and they’ve probably only touched their own skin never mind someone else’s “ (P2)* *"I’ve got a background in massage therapy and I have been doing that for gosh eight years now, so I find some techniques easier compared to other students" (P3)*
**Need for objectivity**	**Theme 5** **Lack of reliability**	**Tutor disagreement**	*“I think there’s a bit of confusion between the tutors and how they do things. One would put the hand below the pelvis and one would put it above the lower back for the same technique. I thought to start with that if at least the three tutors agreed, or four tutors, agreed on what they’re teaching us and teach us this at least there’s no like, yeah but you can do it like this” (P4)* *"Often we have two tutors or more. what is interesting at times is that they all have different ways of doing the same thing and expect us to do it the way they would do it. It can confuse you sometimes and wonder which is the right way to do the technique" (P8)*
		**Self doubts**	*“Yeah the palpation or even when getting the tests done to see how it feels, from someone who feels confident in doing it, whereas us students, you know you’re always wondering ‘oh am I doing it right?’ or if things are a bit fiddly” (P3). "When I started doing this the tutor who was in charge came back to me and said, no that’s wrong. That’s not what you are meant to do. It took me weeks, maybe a month to figure out that if I do with my hands something like this it makes the movement. It makes the actual movement I’m meant to do" (P7)*
	**Theme 6** **Technology enhances confidence**	**Practicing the right thing**	*”… it would definitely be helpful to have something that means you’re more confident and that you’re practising the correct thing when you don’t have the tutor right there. So if there’s some way that it can help it would, yeah. It would be quite helpful” (P3)* *"But I think something that really does need to happen is we almost need to feel and practice the right techniques first. So, technology may help here" (P4)* *"I might be wrong and maybe with the right technology we could go a bit faster and learn more and more precisely and maybe not make some mistakes that I’m still making" (P7)*
		**Haptic feedback**	*“It would be really interesting if you could use your hands to move and manipulate the body or even just to touch it, to highlight a particular muscle or muscle group… perhaps it would make it feel a whole lot more real to me” (P1)* *"I have used VR as part of gaming, some of them come with controllers that even vibrate as you play. It will be interesting if we can have something like that" (P6)* *"We’ve just been doing some stuff in general medicine at the hospital and they’ve got these cool models that you can do stuff on and you get feedback. There’s some sort of electronic thing and it looks like a mannequin of some sort but it’s got all sorts of sensors in it" (P10)*
		**Visual cues**	*"We used visible body, it’s a fantastic 3D anatomy tool and it’s like a human atlas where you can, where you can like directly dissect certain muscles and you can be like cool this where it’s originated attachment is in movement and things like that, and that’s a fantastic tool, you can kind of rotate the body and remove superficial muscles to then see the deep muscles and the underlying structure" (P4)* *“The reason I thought this would be really cool is because the first thing I thought of is if I can see something… then I can sort of match where I’m going. That’s where things stick for me. I don’t know what kind of VR technology you have but if you could see that’s the skin but then under it you’ve got a layer of bones or whatnot then you can match up. I think that would be really good for a lot of visual learners… I think it would be quite powerful in learning” (P8)*
**Tutor Feedback**	**Theme 7** **Left on your own**	**Tutor unavailable**	*“Often we then break off into little groups or pairs to practise it but then it’s very hard for that one tutor to get round all of those pairs to make sure they’re doing it correctly. And often people have questions and then they go caught up talking, so a lot of the time you might be trying to do one practical thing” (P3)* *"I feel like there’s a lot missing in my learning so I have to push myself to learn that stuff" (P9)*
		**doesn’t make sense**	*“The way they explain things don’t make sense. It might not be the best example but we have to manipulate the cervicals. She said you do it like this and she shows. But we can’t even see the fingers underneath the neck so it’s a bit complicated for us”(P6)* *"Often what they say is difficult for us to understand. Other than that really it’s just practise and just asking for help all the time and sort of fake it till you make it. It’s going to be just practise until you feel something hopefully. It takes quite a long time…" (P10)*
	**Theme 8** **Struggles with 2D technology**	**Powerpoint**	*“Everything I’m trying to learn is *via* a PowerPoint or videos which can be challenging. I’m a practical learner so if we don’t do the practical, I struggle in connecting stuff where others pick it up quite well. I don’t learn that way” (P8)* *"PowerPoint and YouTube videos are the only technology used in our course" (P1). "PowerPoint is the only digital technology that am aware of. The tutor starts with PowerPoint and may show videos too, but otherwise, no technology is used" (P6)*
		**Complicated 2D pictures**	*“Apart from PowerPoints and a few videos or something like that is about the most digital we get I think” (P9). Often, we learn from books that describes a technique, hand positions, *etc*. However, I simply cannot learn from 2D pictures. [P10]* *"You know when you’re learning from 2D images the majority of the time, or sometimes like have an actual model, which, you know which is amazing but it’s very generic you know". (P2)*
		**Lack of 3D technology**	*"I feel like they haven’t moved that further, obviously like there’s like an anatomy app that you can do 3D visualisation which I’m using a lot of the time". (P2)*
	**Theme 9** **Hands-on support**	**Cannot replace face-to-face**	*“He gave us specific landmarks that we would look for. For example, C7 would be the most prominent one that sticks out in forward neck flection and that was a good baseline in order to help us when we would palpate for cervical spine. He would give us points in which they were quite useful for our learning” (P5). "Technology would be good, but the tutors provide specific instructions and hands-on real time feedback. So, I think you can’t replace that face to face learning" (P7)*
		**Hands under hands**	*“One thing I found effective with a clinical supervisor I have is we had a patient and he put his hands on top of my hands and helped me to feel the pressure I should be applying, or how I should perform a massage stroke. And that was just like sort of mind-blowing for me” (P1)* *“…we were doing a technique called ‘functional’ on the like upper thoracic and the person is lying down, and you like put your hands under and she comes along, she [tutor] came along, and put her hand under our hand and so she could feel where it needed to go and stuff, so that was really good” (P7)*
**Knowledge**	**Theme 10: knowledge about 3D technology**	**No knowledge**	*“No no, apart from what you've seen on TV with their show these things”. (P4)* *“I don’t even know what it is”. (P6)* *“I have never used such technologies before and have no knowledge about these technologies. We have not been introduced any such technologies as part of the course either.” (P10)*
		**Used as a gaming tool**	*“I have used VR while playing games. So I know what it can offer to us and our learning and looking forward to using it in the future for our clinical practice” (P7)* *“I have used the goggle before for playing computer games. That is how far my knowledge goes but do believe that it will be excellent to have that support as part of our education”. (P5)* *“In terms of virtual reality, no, I’ve only um experienced that in terms of playing games.” (P3)*
		**Previous work**	*“I did, when I was in engineering I used to work for Jaguar Land Rover and they had a thing called a cave, and basically it was like they have a, the geometry of the car, like a finished car and like a, let’s just a Range Rover Discovery and you could actually, this cave was like had I think it was or red walls or whatever it was, you’ve got the VR headset on you can actually go into the car and you can have what the product would look like which was, yeah it was pretty amazing” (P2)*
**Barriers and enabler**	**Cost**		*“I doubt they’ll let us as students take one home because they’re so expensive. If institutions can pay for it so we students can still access it a reduced cost perhaps” (P8)* *“Obviously that financial one may play a big part so I guess having one of those facilitators such as a support might be necessary” (P5)*
	**Knowledge about technology**		*“…People’s knowledge in the tech, around the technology as well if it would be something that people don’t work with a lot and they’re being bombarded with a whole lot of other new information… If it doesn’t work properly the first time it can be rather annoying, or discouraging, would be another barrier to it” (P3)* *“Yep absolutely, if there was adequate training I would be very open to using it yeah.” (P2)*
	**Accessibility**		*“… [I] don’t know how long you can stay in goggles like this before it gives you a headache” (P6)* *“ [I] think one thing is that I can get fatigue from being on technology for a while” (P3). “Maybe internet connections. I don’t know if you need to internet to download specific stuff” (P5).”Connections, issues, are a big one, I’ve noticed that my connections been shocking so it kind of breaks in and out throughout classes which is tricky. Maybe attitude towards technology, I personally, I don’t mind using it but I know some people don’t enjoy using technology, and I guess accessibility for some people as well” (P4)*
	**Ethical issues**		*“There’s got to be like a level of cultural responsiveness or just appropriateness or ethics what would need to, student would have to get. Or it would just have to, the simulation would have to have restrictions” (P1)* *“…the only thing I can think of is like consent and making sure people are okay with what they’re seeing and also by enabling limiters so students” (P2)*

### Findings

Six factors were identified which appeared to influence participants’ perception and knowledge of the role of technology in MT education. These were:

1. The sufficiency of current teaching methods,
2. Evolution as a learner (a novice to an expert),
3. Need for objectivity,
4. Tutor feedback
5. Knowledge and
6. Barriers and enablers.

### The sufficiency of current teaching methods

The sufficiency of current teaching method was a key factor contributing to participants’ perception about the role of technology in MT education. Participants who felt that their current teaching was sufficient believed that technology had no or little role in MT education.

*“I can’t think of another way of doing it than the way they do it. To me what they do is great but it’s maybe because I’ve never thought about [it]” (P6).*

*“I feel like it is sufficient…for us to be a better therapist [and] to learn better with our hands” (P2).*

Participants who perceived the current teaching of MT to be sufficient tended to be osteopathic students learning MT from year one of their program. Being exposed early to hands-on MT courses may have influenced their perception and embedded an established routine for learning.

*“[The] actual practical part side of it I think is really good, like they have a routine. They’ll show us a technique or like a special test or whatever they do, and then we’ll go away in our little groups and practise that, and I think that’s really like effective. Then the teachers come round and like adjust us if needed and just like help us and give us like random tips and stuff” (P7).*

Conversely some participants perceived that their current teaching of MT was insufficient and inadequate.

*“I’d like to say yes but no, not really. I almost feel as if a lot of it is just left up to you to try and work out if you’re doing it right. The tutor can’t feel exactly what you’re doing. They can only look and think it does look you’re pressing too hard or light or whatever” (P9).*

Participants who felt that the MT teaching was insufficient were most likely enrolled in a blended learning pathway where they are on campus for block teaching weeks and off-campus during the rest of the learning period. This model of delivery meant that a lot of content was taught in a short period of time. This made them feel that they lacked time to reflect on their learning and made them feel completely ‘overwhelmed’ while trying to grasp the complex MT skills.

*“The way it’s been working is we have these eight-hour long days and we have to cram/study all this information in the morning, have lunch, and then come back and cram the rest of the knee and maybe even the whole lower limb for the rest of the day and we just go away at night just feeling so overwhelmed, and then write an essay on a completely different subject” (P1).*

### Need for objectivity

The need for objectivity appeared to contribute towards the perception of participants regarding the role of technology in MT education. Some participants felt that the current teaching paradigm was subjective and lacked reliability with different tutors teaching different things.

*“I think there’s a bit of confusion between the tutors and how they do things. One would put the hand below the pelvis, and one would put it above the lower back for the same technique. I thought to start with that if at least the three tutors agreed, or four tutors agreed, on what they’re teaching us and teach us this at least there’s no like, yeah but you can do it like this” (P4).*

The use of different approaches by different tutors and an apparent lack of objectivity lead to self-doubt among some participants about their ability and the correct method.

*“Yeah, the palpation or even when getting the tests done to see how it feels, from someone who feels confident in doing it, whereas us students, you know you’re always wondering ‘oh am I doing it right?’ or if things are a bit fiddly” (P3).*

These participants perceived that technology therefore could enhance their confidence by negating subjectivity.

*“… it would definitely be helpful to have something that means you’re more confident and that you’re practising the correct thing when you don’t have the tutor right there. So, if there’s some way that it can help it would, yeah. It would be quite helpful” (P3).*

Participants who required measurements or an objective way to do things were more likely to believe that technology such as VR is required as part of MT education. They were more likely to perceive that VR would guide them to palpate the structure that they need to, thereby improve the accuracy of palpation.

*“It would be really good if there was some way that you could actually have some sort of measurement of exactly how you do it or whether you’re doing it right or what you’re feeling is the correct thing” (P9).*

In turn, this kind of learning experience is made authentic by enhancing fidelity (realness) for the learners. When presenting examples of the use of VR and how this might enhance understanding and ability to perform skills completely, they responded:

*“The reason I thought this would be really cool is because the first thing I thought of is if I can see something… then I can sort of match where I’m going. That’s where things stick for me. I don’t know what kind of VR technology you have but if you could see that’s the skin but then under it you’ve got a layer of bones or whatnot then you can match up. I think that would be really good for a lot of visual learners… I think it would be quite powerful in learning” (P8).*

*“It would be really interesting if you could use your hands to move and manipulate the body or even just to touch it, to highlight a particular muscle or muscle group… perhaps it would make it feel a whole lot more real to me” (P1).*

### Evolution as a learner (a novice to an expert)

The perception of the role of 3D technology in MT education depended on the expertise level/evolution of the learners. Participants who were early on in their educational journey felt that the current teaching methods (see one, do one approach) to be adequate. They were comfortable learning gross motor skills (e.g., holding a leg) that does not require deeper palpation skills.

*“At the moment because I’m a first year I’m getting comfortable with touching people and making sure that when you’re holding their leg they feel like she’s got me. That’s what we’re working on at the moment” (P6).*

Conversely, participants who were at the later stage of their educational journey emphasized the need for technology to support the development of finer motor skills required for deeper and subtle palpation.

*“I’m finding it really difficult because you don’t know what you’re trying to feel. Don’t know what you’re feeling for and trying to translate a description into trying to work out what I’m feeling. I’m actually struggling with that a little bit. Generally, the more obvious techniques are great but when it comes to really subtle palpation it’s really difficult to try and understand what you’re meant to be feeling for” (P9).*

It was noted that participants’ who had already completed MT courses before were likely to perceive that the current teaching methods were adequate and the need for technology to be minimal. Participants with MT experience felt that students who have not done MT before would require more support.

*“I have worked in massage now for five years, so I’ve got a lot of palpatory hands on experience but it doesn’t make me like an osteopath or anything, but for some of the school leavers, they come straight out of school and they’ve probably only touched their own skin never mind someone else’s” (P2).*

### Tutor feedback

A key factor mandatory for learner development is feedback from tutors. Most participants felt that the current teaching methods were inadequate and unsustainable as often there is often only one tutor running a teaching session. This meant that they did not receive enough feedback where they could refine their MT skills.

*“Often we then break off into little groups or pairs to practise it but then it’s very hard for that one tutor to get round all of those pairs to make sure they’re doing it correctly. And often people have questions and then they go caught up talking, so a lot of the time you might be trying to do one practical thing” (P3).*

*“The way they explain things don’t make sense. It might not be the best example, but we have to manipulate the cervical [spine]. She said you do it like this and she shows. But we can’t even see the fingers underneath the neck so it’s a bit complicated for us” (P6).*

Participants that received less tutor feedback explained that they were trying to learn complex MT skills from 2D images or PowerPoint presentations which can be challenging. Hence, they perceived that 3D technology such as VR would be important in enhancing their leaning.

*“Everything I’m trying to learn is via a PowerPoint or videos which can be challenging. I’m a practical learner so if we don’t do the practical I struggle in connecting stuff where others pick it up quite well. I don’t learn that way” (P8).*

*“Apart from PowerPoints and a few videos or something like that is about the most digital we get I think” (P9).*

On the contrary, some participants felt that they received good feedback from their teachers, which meant that they relied less on technology.

*“He gave us specific landmarks that we would look for. For example, [the] C7 [spinous process] would be the most prominent one that sticks out in forward neck flexion and that was a good baseline in order to help us when we would palpate for [the] cervical spine. He would give us points in which they were quite useful for our learning” (P5).*

Specifically, these participants felt that they learnt more when the tutors placed their hands on them and showed them how to do a certain technique. This human interaction therefore was key in learning MT.

*“One thing I found effective with a clinical supervisor I have is we had a patient and he put his hands on top of my hands and helped me to feel the pressure I should be applying, or how I should perform a massage stroke. And that was just like sort of mind-blowing for me” (P1).*

*“…we were doing a technique called ‘functional’ on the like upper thoracic and the person is lying down, and you like put your hands under and she comes along, she [tutor] came along, and put her hand under our hand and so she could feel where it needed to go and stuff, so that was really good” (P7).*

### Knowledge

Knowledge about technology was an important factor that determined the acceptance of 3D technology as part of MT education by students. Some participants did not have any knowledge about 3D technologies such as VR.

*“I have never used such technologies before and have no knowledge about these technologies. We have not been introduced any such technologies as part of the course either.” (P10)*

Some participants had previous knowledge about 3D technologies, however, their knowledge about 3D technology was confined to playing games.

*“In terms of virtual reality, no, I’ve only um experienced that in terms of playing games.” (P3)*

The exposure, knowledge and positive experience with VR games made these participants to believe that technologies such as VR may support their clinical education and practice.

*“I have used VR while playing games. So I know what it can offer to us and our learning and looking forward to using it in the future for our clinical practice” (P7).*

*“I have used the goggle before for playing computer games. That is how far my knowledge goes but do believe that it will be excellent to have that support as part of our education”. (P5).*

Few participants had good knowledge about VR as it was part of their previous education and/or work experience.

*“I did, when I was in engineering I used to work for Jaguar Land Rover and they had a thing called a cave, and basically it was like they have a, the geometry of the car, like a finished car and like a, let’s just a Range Rover Discovery and you could actually, this cave was like had I think it was or red walls or whatever it was, you’ve got the VR headset on you can actually go into the car and you can have what the product would look like which was, yeah it was pretty amazing” (P2)*

### Barriers and enablers

Some factors were both barriers and enablers for using 3D technologies as part of MT education. These factors include (1) cost, (2) knowledge about technology, (3) accessibility, and (4) ethical issues.

Almost all the participants felt that cost was a significant barrier if technology such as VR to be used in MT education.

*“I’m sure that the cost of it is one main problem. People even thinking about it. People developing it and being paid to develop it and then people would have to buy that technology” (P6).*

*“The cost. I think that would be a big thing would be the cost really. That would really be the only barrier that I can think of” (P9).*

The participants believed that the cost on students could be reduced if the institutions could bear some or most of it thereby enabling students to access technology.

*“I doubt they’ll let us as students take one home because they’re so expensive. If institutions can pay for it so we students can still access it [at] a reduced cost perhaps” (P8).*

*“Obviously that financial one may play a big part so I guess having one of those facilitators such as a support might be necessary” (P5).*

All participants indicated that they would be keen to try technology if it is available. However, some required further knowledge about technology. They thought that using technology without completely understanding it or if the technology did not work properly, may discourage them from using it.

*“…People’s knowledge in the tech, around the technology as well if it would be something that people don’t work with a lot, and they’re being bombarded with a whole lot of other new information… If it doesn’t work properly the first time it can be rather annoying, or discouraging, would be another barrier to it” (P3).*

*“Yep absolutely, if there was adequate training, I would be very open to using it yeah.” (P2).*

Some participants thought that perceived ease of access could a barrier from using technology such as VR. They felt that using technology for a long time can lead to headache and fatigue and discourage further use.

*“… [I] don’t know how long you can stay in goggles like this before it gives you a headache” (P6).*

*“[I] think one thing is that I can get fatigue from being on technology for a while” (P3).*

In terms of ease of access, internet connection was identified as another barrier.

*“Maybe internet connections. I don’t know if you need to [use] internet to download specific stuff” (P5).*

*“Connections, issues, are a big one. I’ve noticed that my connections been shocking so it kind of breaks in and out throughout classes which is tricky. Maybe attitude towards technology, I personally, I don’t mind using it, but I know some people don’t enjoy using technology, and I guess accessibility for some people as well” (P4).*

A few participants were concerned about ethical issues such as cultural responsiveness that may arise using technology such as VR as part of MT education.

*“There’s got to be like a level of cultural responsiveness or just appropriateness or ethics what would need to, student would have to get or it would just have to, the simulation would have to have restrictions” (P1).*

On the other hand, these ethical issues could be overcome by completely explaining the design of VR.

*“…the only thing I can think of is like consent and making sure people are okay with what they’re seeing and also by enabling limiters so students” (P2).*

**Fig. 1:**
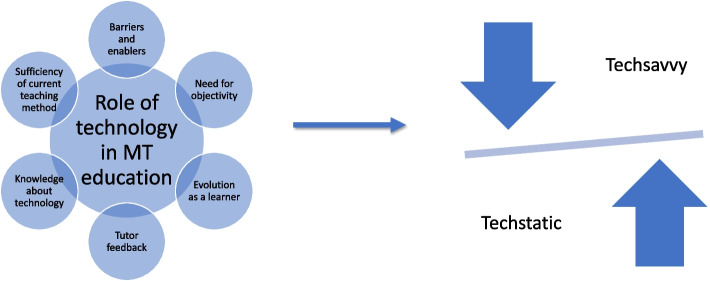
Factors influencing and associated with participants’ perception of role of 3D technology in manual therapy education

The six factors discussed above in turn influenced the participants’ perception and knowledge (which seems to be intertwined) about the role of 3D technologies in MT education with participants evidently taking two distinct/polarized positions (‘no role’ (techstatic) versus a ‘complete role’ (techsavvy)). Figure 1 depicts the interplay between the five factors and how they influence the learner’s position as either being techsavvy or being techstatic.

Participants who perceived that technology had no role in MT education tended to view that the current (“see one, do one”) approach was best suited for MT education.

*“I think the current way manual therapy is taught is sufficient. I don’t know how else they could do it really. Just putting your hands on and getting used to that and getting better palpation, you can do that through practise” (P7).*

These participants believed that technology cannot replace human interaction and that ‘face to face’ sessions are important to learn the complex MT skills.

*“Technology has a place, but again I think you can’t replace face to face learning. I think there needs to be that sense of community still, like there’s nothing better than being in class with our classmates” (P4).*

With a strong emphasis on human interaction, they conceptualised MT education as an approach that should involve “hands-on hand” feedback. One participant explained this as:

*“He put his hands on top of my hands and helped me to feel the pressure I should be applying, or how I should perform a massage stroke. And that was just like sort of mind-blowing for me because I was like ‘oh that’s how you do it, that’s how it’s supposed to feel, that’s what you’re doing’ and I could sort of feel through my hands” (P1).*

Participants on the opposite end of the continuum perceived technology as a ‘futuristic’ and an ‘advanced’ way to learn MT.

*“…if you had goggles on and you had a fake patient in front of you, how you would be able to see everything quite clearly and if they had designed some cool gloves then maybe you could actually feel what it would feel like to touch the patient… that’s going way advanced” (P2).*

These participants believed that the repeated practice opportunity that technology offers, provides an ‘objective’ way to learn MT skills.

*“Oh totally, yeah big time. Now I think it’s just a matter of time. I think technology such as VR is futuristic and may help learn things objectively” (P10).*

*“Technology is awesome. I think most of our learning in manual therapy is self-directed…technology will provide repeated practice opportunity given that we can use them [technology] at home” (P6).*

## Discussion

The findings from our study indicate that MT learners have different views about the sufficiency of current teaching methods, their evolution as a learner, need for objectivity, tutor feedback, knowledge, and barriers and enablers, which together shape their overall perception about the role of technology in MT education. Such diverse viewpoints amongst MT learners are consistent with learners from other medical professions.

Learners who perceived that technology has no or minimal role in MT education alleged that the current way of teaching MT was adequate and the ‘see one, do one’ approach is the best way to deliver this form of education. These participants considered their teachers were effective, had excellent teaching skills and respected them as students. By sharing real life scenarios and demonstrating hands-on techniques, these teachers were ‘preferred’. Although “teacher-centred”, preferred teachers had a positive impact for these learners and motivated them in learning MT skills. Hence, the teaching style and the positive interaction that they adopted were considered sufficient by participants. This is synonymous with the findings of previous studies that showed that ‘preferred teachers’ have a critical influence on learner’s academic success, professional and personal development [36, 37]. Further, these participants perceived that face-to-face teaching was crucial for their learning and ‘nothing could replace human interaction’ [38].

In opposition, some participants felt that technology has a bigger role in MT education and may provide objectivity for their learning. These participants were likely to perceive that the ‘see one do one’ approach has its limitations and current methods for teaching MT was insufficient. These findings concur with that of a randomised controlled trial [10] that compared a ‘best practice’ model with the traditional model of teaching. While the ‘best practice’ model included structured feedback, practice on manikins and the Peyton’s ‘four-step’ approach; the ‘traditional model’ was the ‘see one do one’ approach. The study showed that the traditional ‘see one do one’ approach had limitations and the ‘best practice’ model resulted in students performing not only in the short term but also in the long term [10].

Tutor feedback was identified as a key factor that determined the perception of participants regarding the role of 3D technology in MT education. Our findings suggest that participants who received less feedback from their tutors perceived that 3D technology such as VR would be important in enhancing their leaning. We suggest that this is not surprising given the difficulty associated with learning complex MT skills. These findings are consistent with previous evidence that feedback assists medical students to get an understanding/feeling for what they do and increases the likelihood of correct performance [39, 40]. Some participants believed that they get satisfactory feedback from tutors either through role play or tutors who placed their hands on top of theirs and showed them how to undertake a certain technique. However, evidence dispute these claims reporting that such learning (see one do one) may miss essential components such as self-regulated learning, review at each stage and self-monitoring resulting in less retention of the skill set in the long term [7, 8].

Lack of opportunity to practice was also highlighted by participants as a limitation of current way MT is being taught. This led those participants to believe that having access to technology may facilitate practice/repetition, which in turn may facilitate their hands-on skill development. These findings concur with previous research which clearly indicates that practice (or lack of it) can influence student outcomes [41]. Also, a lack of practice may have serious implications in terms of patient safety and therefore an insurance risk for educational institutions [4]. Therefore, 3D technology such as VR may provide a fail-safe environment and an opportunity for repeated practice for students could be a worthwhile investment [13]. It is important to note that technology alone (e.g. simulator training) is not enough to improve learners skill performance, and feedback from an expert teacher is also important to enhance their skill performance [10].

Our findings suggest that the expertise level of a learner was an important factor that influenced the perception of role of 3D technology in MT education. Participants who were early on in their educational journey were comfortable without the aid of technology while learning gross motor skills (e.g., holding a leg) that does not require deeper palpation skills. Alternatively, participants who were at the later stage of their educational journey perceived that technology would be essential to support the development of finer motor skills/complex tasks required for deeper and subtle palpation. In this context, a complex task is one that requires: long reaction time or movement time, long hours of practice and high demands on the learner’s attention and memory [42]. Evidence suggests that 3D technologies such as VR may enhance the development of complex skills by providing abundant practice repetitions, delivering multi-sensory feedback, individualize challenges, and engage and motivate users with salient, enriched environments [43]. Our findings are therefore consistent with of the previous studies that have shown 3D technology such as VR may improve both gross and fine motor skills [44–47].

Knowledge about 3D technologies was identified as an important factor that would determine leaner’s acceptance of such technologies as part of their MT education. The common 3D technology that the participants reported using (or being used by lecturers) was 3D anatomical models and/or applications such as ‘visible body’ to learn human anatomy. This is in agreement with previous findings from medical education research [48]. Importantly, none of our participants reported using or being exposed to 3D technologies such as VR/AR as part of their education. As far as we know, there are no undergraduate MT programs that utilize VR or AR as part of MT training, which is in agreement with previous findings [28]. Some participants had exposure to 3D technologies such as VR outside of their MT program/educational institution in the form of virtual games. This exposure to VR in turn may have made these participants to have an open attitude and were likely to utilize such technologies if offered. On the other hand, participants who did not have knowledge about and/or exposure to 3D technologies were likely to be conservative in incorporating these technologies as part of their MT education. Both the enthusiasm of participants with prior knowledge of 3D technologies and the resistance of those who did not have much knowledge corroborate with those noted in other studies [48–50].

Several factors were identified as barriers and enablers for using 3D technologies as part of MT education including cost, knowledge about technology, accessibility, and ethical issues, which are consistent with the existing literature [51–56]. Key barriers include concerns about hardware devices such as head mount devices and the time required to learn the technology [56–59]. Addressing these barriers may require a collaborative approach from clinicians and developers to meet the specific demands of MT education [60]. For example, the physical assessment/treatment parameters required for a knee joint will be different from that of neck and so on. As highlighted by a recent scoping review [28], MT educators may share the clinical reasoning behind a physical assessment procedure enabling the developer to integrate the software parameters that control the degree of physical tasks and challenges to meet the assessment/treatment needs. This step may be crucial to sustain the motivation and engagement of learners over a longer period [22].

To summarise, the views of our participants were polarized with some considering the current teaching model (see one, do one) to be sufficient, whereas other participants considering technology to replace current teaching methods. Considering both perspectives, it could be argued that the “see one, do one” approach of learning/teaching is still applicable as human interaction is important; however, there is scope to build upon and enhance this with various other learning principles and advanced technology. This is consistent with the findings of a recent study which found that although students perceived distance learning to be good, they were not satisfied with this type of learning. Potential predictors for learner’ satisfaction of distance learning included instructor support for students, personal relevance, previous experience in distance learning, and being a master's student [21].

According to contemporary educational theory, learning happens in a zone referred by Vygotsky as the ‘zone of proximal development’ [61]. Practising beyond these limits without support is similar to practising with increased stress, less confidence and marginal competence [61]. This is considered harmful as this is the zone where learners are not capable and/or not ready for doing things [61]. Yet, this is the zone that learners encounter often with the ‘see one, do one’ methodology, especially in the last 2 years where class disruptions due to the COVID-19 pandemic were frequent and face-to-face contact has been minimal. Hence, to ensure that the learners stay competent, strategies in addition to current teaching methods may be required. Using 3D technologies such as VR to complement current teaching methods may represent such an additional strategy and may decrease extraneous stress on the leaners. Future research may investigate the addition of 3D technology to traditional teaching methods in improving MT assessment/treatment by MT learners.

### Strengths and limitations of the study

To our knowledge, this is the first qualitative study to explore the perceptions of MT learners on the role of 3D technologies in MT education. The main strength of this study was that it was open to all MT students despite the discipline that they were training in (e.g., physiotherapy, osteopathy, etc.). The participants came from different disciplines (physiotherapy and osteopathy), from different pathways of learning (traditional vs blended learning), and different years of learning (first year through final year of learning). This variety in participants resulted in rich data that provided interesting perspectives on the role of 3D technologies in MT education. We followed a robust protocol to reduce bias and enhance credibility of the findings and used the COREQ guidelines to improve transparency in reporting [30]. Currently, we are not aware of any MT program that employs 3D technologies as part of their teaching curricula. Hence, the views of participants expressed in this study reflected their perceptions, knowledge, and exposure of/to such technologies. However, understanding the learner’s perspective is important before such technologies are offered as part of their education.

The study is not without its limitations. A key limitation is that all the participants were learners of MT in New Zealand institutions. Hence, the transferability of findings to MT learners in other countries needs to be established through future research. Despite our best efforts, we did not have any participants from the chiropractic profession. However, our data has captured different perspectives and may be applicable to other professions that uses MT. Future studies in this area on students, clinicians and academics from different professions using MT from low- and high-resource settings are warranted. Finally, it could be argued that the nature of program (continuous or blended) may affect learner’s perception. However, on data immersion and repeated reading of the transcripts, most challenges experienced by learners seem to be consistent despite their program structure. For example, sufficient teaching methods, good feedback, and objectivity are common expectations of these learners.

According to a recent scoping review, there is no specific tool (augmented, virtual, or mixed reality application) readily available for teaching manual therapy (e.g., joint motion assessment) [28]. However, the available applications [29, 62] can be easily adapted to train skills of tissue palpation in the future studies. The follow-up studies may investigate the perceptions, attitudes, MT skills, and safety concerns of the learners and teachers following their exposure and training with culturally and ethically appropriate digital technologies (e.g., applications with VR).

## Conclusion

Participants in this study held a range of views regarding the role of 3D technologies in MT education. Five factors were identified to influence learners’ perception: the sufficiency of current teaching methods, evolution as a learner (a novice to an expert), need for objectivity, tutor feedback, and barriers and enablers. These views and perceptions contributed to two opposing positions “techstatic” or “techsavvy”. However, technology may be used to complement the traditional “see one, do one” approach of learning/teaching to facilitate the learning of complex skills by MT learners. The advantage of such an approach is an area of future research.

## Supplementary Information

**Additional file 1.**

## Data Availability

The datasets generated and/or analysed during the current study are not publicly available due to the qualitative nature of the study but are available from the corresponding author on reasonable request.
