# Supplementary material for: Perception and knowledge of learners about the use of 3D technologies in manual therapy education – a qualitative study
Source: BMC Med Educ. 2023 Jul 17;23:509. doi: 10.1186/s12909-023-04497-6 (PMC10351149; doi:10.1186/s12909-023-04497-6)
Supplement: Supplementary file 1 — Additional file 1. [file 12909_2023_4497_MOESM1_ESM.pdf]

Interview guide (for students)

Pre-briefing statement guide

I am [name], an [role: researcher/academic) from [X institution], and working as part of a research team exploring the role of 3D digital technologies such as virtual reality, augmented reality, and mixed reality in manual therapy education.

Information about the research is detailed in the information sheet that was sent to you in the email. Hope you had a chance to read the information sheet. Do you have any questions about the project?

Thanks for agreeing to be interviewed. I want to emphasise that everything we discuss will remain confidential within the research team. If we report on anything you say it will be fully anonymised, so that no one could trace comments back to you.

There are no right or wrong answers to our questions; we are interested in your personal views, experiences and perceptions. So please be as honest and open as you can be, as this will help us understand the usage of virtual reality as part of your education. Is that all clear? Do you have any further questions before we begin the interview?

Opening questions

You filled in the demographic form, so you… [use as a prompt for an introductory narrative/conversation about their background as an educator or student].

1. What led you to become a physiotherapy/osteopathy/manual therapy student? [And what have been the biggest influences on you as a student?]

2. Why did you choose to take part in the study?

Views on current teaching/learning paradigm of MT

3. How would you describe your current teaching of MT?

4. What does being a MT student mean to you?

5. Do you think/believe that your current paradigm of MT teaching is sufficient for you as a learner to learn the complex skills required for doing MT? if yes, in what ways, if not, what needs to improve?

 [Prompt, for example, palpating the spine]

Current usage of technology in MT teaching/education

6. Can you briefly take me through your last MT teaching session?

7. Thinking of specificity, can you describe what framework/strategy your educators used to ensure that you are assessing/palpating the structure that you are supposed to.

9. Did your educator or institution use any technology as part of your MT learning? If yes, please expand.

Attitudes towards technology of MT teaching/education

10. When I say '3D technologies' what comes to your mind?

11. What is your perception/experience of 3D technology such as VR or AR in general?

12. Do you think technologies such as VR or AR have a role in MT education?

13. If given an opportunity, would you explore/try 3D technology as part of your MT learning? If yes, expand more, if not, why not?

Barriers and Facilitators

14. What do you think are the factors that may facilitate the use of 3D technologies in MT education?

15. What do you think are the barriers for the use of 3D technologies in MT education?

16. Is there anything else you would like to add?

End.
